# Across‐Fault Velocity Gradients and Slip Behavior of the San Andreas Fault Near Parkfield

**DOI:** 10.1029/2019GL084480

**Published:** 2020-01-17

**Authors:** N. Piana Agostinetti, G. Giacomuzzi, C. Chiarabba

**Affiliations:** ^1^ Department of Geodynamics and Sedimentology University of Wien Wien Austria; ^2^ Istituto Nazionale di Geofisica e Vulcanologia Rome Italy

**Keywords:** Fault rheology and slip behavior, acrosso fault velocity gradients, fully non‐linear tomography

## Abstract

A long‐lasting question in earthquake physics is why slip on faults occurs as creep or dynamic rupture. We compute passive measurements of the seismic *P* wave velocity gradient across the San Andreas Fault near Parkfield, where this transition of slip mode occurs at a scale of a few kilometers. Unbiased measurements are obtained through the application of a new Bayesian local earthquake tomographic code that avoids the imposition of any user‐defined, initial velocity‐contrast across the fault, or any damping scheme that may cause biased amplitude in retrieved seismic velocities. We observe that across‐fault velocity gradients correlate with the slip behavior of the fault. The *P* wave velocity contrast decays from 20% in the fault section that experience dynamic rupture to 4% in the creeping section, suggesting that rapid change of material properties and attitude to sustain supra‐hydrostatic fluid pressure are conditions for development of dynamic rupture. Low *Vp* and high *Vp*/*Vs* suggest that fault rheology at shallow depth is conversely controlled by low frictional strength material.

## Key Points

- Velocity gradients across the SAF (differences in *Vp* and *Vp*/*Vs* between the two sides of the fault) correlate with the slip attitude
- This finding has great impact for understanding earthquake physics and help creating bridges over disciplines and scales (nature to labs)
- The newly developed fully nonlinear tomographic technique permits to resolve high details of faults and seismogenic processes

## 1 Introduction

Faults on Earth fail with a continuous spectrum of slip modes, between the end‐member of aseismic creep and earthquakes (Sibson, 1977; Scholz, 1998; Harris, 2017). Results of past decades still do not clarify why some faults slip in stable or unstable mode, although results from laboratory experiments and in situ observation offer a deep understanding of processes (Di Toro et al., 2011; Dieterich, 1979; Kanamori & Brodsky, 2004; Marone, 1998 *)*. Dynamic evolution of strength on faults controls the transition of slip behavior (Kaproth & Marone, 2013). The frictional instability evolves, after the onset of sliding, driven by lowering of dynamic friction on the fault plane or change in stiffness in the volume around the fault (Scuderi et al., 2015).

Pioneering work in the early 90s recognized material property changes along faults and proposed relationship with fault rheology (Michael & Eberhart‐Phillips, 1991), and seismic velocity gradients have been proposed as a tool to map potentially hazardous faults in the mid‐crust (Ellis et al., 2017). Recent laboratory experiments and observations in nature (Leeman et al., 2016, and references therein*)* refuel the debate on the role played by physical properties of fault zones.

**Figure 1:**
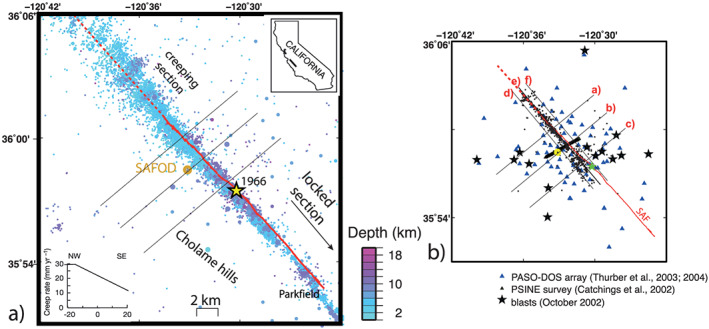
(a) Seismicity in the Parkfield section of the SAF, taken from the NEIC catalog. Relief map of the study area showing the location of the SAFOD drill site and seismicity occurred over the past decades coded with respect to hypocentral depth (see scale). The red line marks the trace of the SAF. The M6.0 1966 epicenter, taken from Thurber et al. (2004), is shown with the yellow star. The traces of sections in Figures 3 and 5 and those used for computing velocity gradients are shown. (b) Data used in the inversion including PASO array (blue triangles), shots (stars), and the PSINE seismic profile (green triangles). The red line marks the trace of the SAF. Seismicity is shown by black points. Section traces used for the vertical sections and to compute velocity gradients across the SAF are shown.

In this paper, we present new measurements of the velocity gradients along the Parkfield section of the San Andreas Fault (SAF), an ideal fault segment characterized by a progressive decrease in creep rate and slip mode change to dynamic rupture proceeding south toward the locked section (Figure 1a). The measurements are obtained through the application of the recently developed full nonlinear local earthquake tomography (LET, based on a reversible‐jump Markov chain Monte Carlo [RjMcMC] sampling; see Piana et al., 2015). This algorithm does not need to impose a user‐defined velocity gradient across the fault or any damping scheme that may cause biased amplitude in retrieved seismic velocities, as in some previous LET applications (e.g., Thurber et al., 2004) and, thus, permits to resolve data‐driven across‐fault seismic velocity gradients along the sections with different slip behavior. The high‐quality data accumulated in this densely instrumented area (Figure 1) are perfect input for high‐resolution imaging and direct comparison with multidisciplinary data and models. The San Andreas Fault Observatory at Depth (SAFOD) drilling project penetrated the SAF at a depth of about 2.7 km, near the southern terminus of the creeping section and NW of Mw 6.0, 2004 Parkfield earthquake hypocenter (Bakun et al., 2005; Zoback et al., 2011). The finding of fault strands with different slip behavior and correlation with weakening processes of material (Carpenter et al., 2015) were used to calibrate our interpretation.

## 2 Data and Methods

Several tomographic models have been published for the Parkfield area using different data set and techniques (e.g., Roecker et al., 2004, 2006; Thurber et al., 2003, 2004; Thurber et al., 2006; Zhang et al., 2009). To ease a direct comparison between results obtained with our new methodology with those from existing models, we use the same data set used in most of them (Figure 1b). The backbone comes from data acquired by the Parkfield Area Seismic Observatory, a dense seismic array of 59 stations operating between 2000 and 2002 around the SAFOD drilling site. Additional data come from the UCBerkeley's High Resolution Seismic Network, the

U.S. Geological Survey CALNET seismic network, the 32 three‐component sensors installed in the SAFOD pilot hole and from 17 shot points, and active seismic data from the PSINE survey (Catchings et al., 2002) and shots in 1994 (Li et al., 1997). The blasts group consists of 66 shots detonated in October 2002 and acquired by the Parkfield Area Seismic Observatory network and borehole sensors and six explosions detonated in 1994. The overall arrival time data set consists of 43948 *P* wave and 29158 *S* wave arrival times, accurately selected to take care of seismic anisotropy (see Text S1 in the supporting information), from 861 earthquakes, 72 blasts, and 82 shots. Each arrival time has been associated with picking quality and data uncertainties have been estimated between 0.025 s (high‐quality arrivals) and 0.3 s (low‐quality arrivals). Double arrivals of *S* phases denoted the presence of seismic anisotropy in the crust (see Text S1). Following a widely used approach, for example, Hua et al. (2017), we retrieve here the isotropic structure of the area, leaving the definition of the anisotropic parameter for future investigations. However, we assume that the spatial distribution of events and stations gives us a seismic ray coverage that prevents the occurrence of strong bias on *Vp*/*Vs* estimations related to anisotropy.

**Figure 2:**
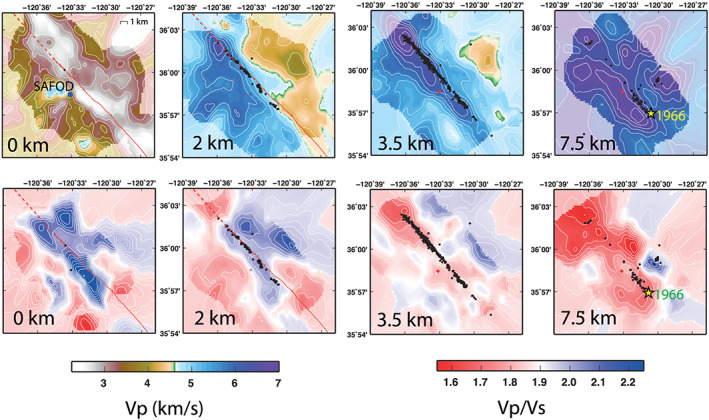
Velocity models along the Parkfield section of the SAF. Vp (top panels) and *Vp*/*Vs* (lower panels) on horizontal cross‐sections at 0‐, 2‐, 3.5‐, and 7.5‐km depths. Relocated earthquakes occurring within ±1 km from the layer is shown. Full color marks regions where the posterior standard deviation is less than the prior standard deviation (well resolved). Note the sharp *Vp* contrast that marks the SAF. Distinct high and low *Vp*/*Vs* anomalies are observed along the fault at different depths.

We use the seismic data to infer the velocity contrast across the SAF, also called “horizontal across‐fault gradient,” by means of a new LET code based on an RjMcMC algorithm (Piana et al., 2015 for details). One of the strong advantages of our new nonlinear technique is that a priori information is less invasive than subjective choices usually required to constrain the inversion in linearized approaches (see examples in Piana Agostinetti et al., 2017). In fact, some of the LET inversions that reconstruct the velocity volume around the SAF imposed a user‐defined velocity contrast across the SAF itself (see Figure S7). Here we only use very loose a priori information on velocity at depth for the whole investigated volume, that is, a simple 1‐D flat prior distribution for velocity parameters (prior standard deviation for *Vp* and *Vp*/*Vs* ratio are 0.5 km/s and 0.15, respectively; see Piana et al., 2015, for details). Given the nature of the prior information used here, any resultant velocity contrast in the sampled models is totally dictated by the information contained in the input data. Indeed, the ability to resolve in 3‐D the SAF as a quasi‐vertical sharp discontinuity (Figure 2) without imposing a priori constraints emerges as the main outstanding results. The geometry and position of the SAF discontinuity, needed to compute across‐fault gradients, is defined here with a precision that accounts for the expected resolution of the velocity model (i.e., within hundreds of meters).

Briefly, our new nonlinear approach follows a workflow that makes it sampling a chain of complete models (i.e., models containing simultaneously both hypocentral, *Vp* and *Vp*/*Vs* ratio parameters) that satisfies the prior knowledge about the elasticity in the study area. Each model is composed of a vector of values for the hypocentral parameters and a vector of values for the position and elasticity of a variable number of Voronoi cells. Voronoi cells are convex polyhedra that form a unique coverage of the investigated volume. The number of Voronoi cells in each model can change along the chain of sampled models. Thus, the sampled models can be composed of a different number of elastic parameters. It is worth noticing that the prior information on the number of Voronoi cells is uniform between 1 and 1500, so the final number of Voronoi cells is uniquely dictated by the data themselves, that is, it is a fully adaptive tomography. For each model, its likelihood to the observed arrival time data sets is computed. Sampled models along the chain are accepted or rejected according to the Metropolis' rule, so that models with worse misfit with respect to the previous one along the chain can still be accepted, even if with limited probability depending on how much the misfit deteriorates. When the chain ends, the family of sampled models forms the target parameter distribution (so‐called “posterior probability distribution” or PPD), and it is analyzed for defining relevant estimators. Here we use the “posterior standard deviation” of the elastic parameters to highlight data‐constrained rock volumes, that is, standard deviations lower than 0.5 km/s and 0.15, for *Vp* and *Vp*/*Vs*, respectively, are considered to define well‐resolved subsurface regions (see Figures S2 and S3). In fact, retrieving a posterior standard deviation lower than the prior standard deviation means that the data give us new constraints about the investigated parameters. It is worth noticing that “intrinsic priors” (see Gao & Lekic, 2018; Hawkins et al., 2019) could be introduced by, for example, the selected Voronoi parameterization or the forward solver. Those priors should be considered when comparing posterior and prior standard deviations, for example, reducing empirically the reference prior value. In the case of the forward solver, “extreme models,” that is, models representing critical (>2.0 km/s) velocity contrast across the fault, could be penalized along the chain by the poor performance of the ray tracer. However, as demonstrated in Figure S4, the across‐fault velocity gradients are still robustly resolved, in the investigated section of the SAF, having posterior std smaller than 0.2 km/s for *Vp* and 0.04 for *Vp*/*Vs* ratio (about 4 times smaller than the prior std for both parameters).

## 3 Velocity Models of the SAF Near Parkfield

### 3.1 Shallow Layers (0–2 km)

The *Vp* structure is dominated by the velocity contrast across the SAF, with the southwest side on average about 1.0 km/s faster than the northeast side (Figure 2). The seismicity is localized on the northern part of the SAF surface trace (red line). NW‐SE elongated low‐*Vp* (<3 km/s) and high‐*Vp*/*Vs* belts to the east of SAFOD and around the SAF trace correspond to shallow tertiary sediments in the Pacific side and highly fractured Franciscan rocks mapped in the North American (NA) side of the fault (see Figure S5). At 2‐km depth, velocity in the range 3–4 km/s corresponds with weathered crystalline rocks and well‐cemented sedimentary rocks (Bradbury et al., 2007; McPhee et al., 2004; Catchings et al., 2002). Two elongated high‐*Vp*/*Vs* belts are found below the Middle Mountain and in the Parkfield grade in correspondence of Franciscan rocks.

### 3.2 Deep Layers (3–10 km)

**Figure 3:**
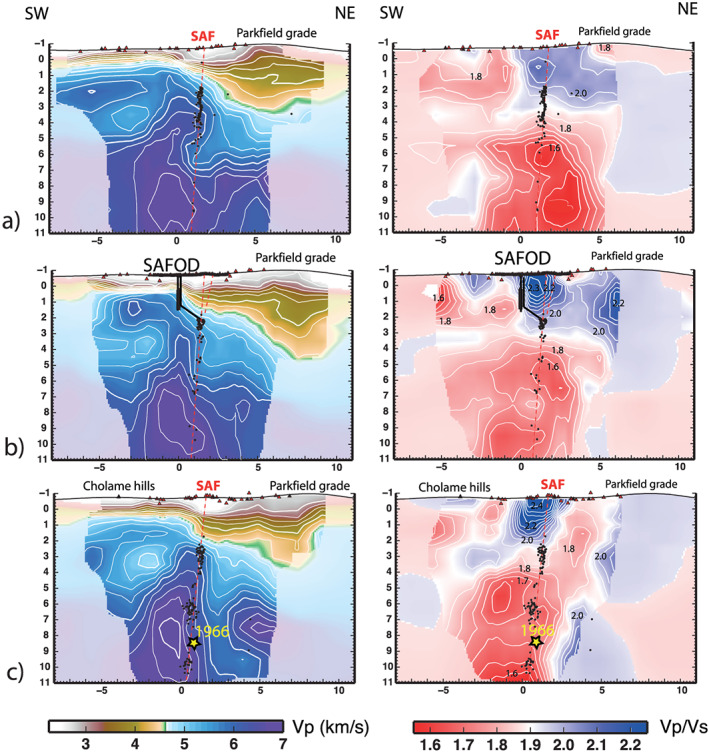
Velocity models across the SAF. Mean *Vp* (left panels) and *Vp*/*Vs* models (right panels) plotted on vertical cross‐sections across the SAF (a–c) (traces in Figure 1). Full‐color regions are areas where the posterior standard deviation is less than the prior standard deviation. Triangles are the used stations; dots are earthquakes hypocenters relocated during the tomography inversion.

The strong velocity contrast across the SAF is visible down to about 10‐km depth where *Vp* of about 7 km/s is found to the southwest of and parallel to the SAF (Figures 2 and 3). The *Vp*/*Vs* is almost everywhere less than 1.75. Although *Vp* and *Vp*/*Vs* models excellently match independently available constraints (see supporting information), absolute *Vp* values in excess of 6.2 km/s suggest the existence of strong heterogeneity within the Salinian composite terrane and mixture of granodiorites with higher‐velocity rocks. The exceptionally high *Vp* and low *Vp*/*Vs* are consistent with rocks metamorphosed to upper amphibolite facies and locally granulite facies intruded by the Mesozoic granitic rocks. In the NA side, the *Vp* sharply decreases to values of about 5.5 km/s, consistent with the extent of Franciscan rocks. Regions of high *Vp* (>6.5 km/s) are present also to the NE of the SAF, at depth greater than 5–6 km. Similar high *Vp* anomalies have been associated with the Permanente Terrane (Thurber et al., 2006).

## 4 Comparison With Previous Models

Several local earthquakes tomographic models exist for SAFOD and Parkfield (Eberhart‐Phillips & Michael, 1993; Lees & Malin, 1990; Roecker et al., 2004, 2006; Thurber et al., 2003, 2004). Despite differences in ray tracing, parameterization, and treatment of earthquake locations, all models show first‐order similarity in the *Vp* structure. The main result is a sharp horizontal *Vp* contrast (up to 1 km/s) across the SAF below 2‐ to 3‐km depth. In some of the previous inversions, this jump was partially related to a priori constraints. Substantial differences appear only in the joint gravity‐velocity model by Roecker et al. (2004, 2006), which shows a velocity reversal to the SW of the SAF in between 3‐ and 5‐km depths.

We observe a first‐order similarity with *Vp* models obtained by previous investigations. In our model, the velocity contrast across the SAF and the low‐*Vp* region to the NE of the SAF, free of a priori constraints, extend to a significantly greater depth and for the entire modeled section of the fault. Our high‐resolution *Vp* model has a very high similarity with active‐source models (Figure S9) in the upper portion of the fault.

A detailed comparison between RjMcMC and linearized inversion results is reported in the supporting information. We observe significant difference in *Vp*/*Vs* models, both across and along the fault (Figure S7). In particular, we resolve a high *Vp*/*Vs* volume in the upper 3 km of the fault that was not imaged in previous papers (Thurber et al., 2004). This shallow high *Vp*/*Vs* anomaly is consistently defined for the entire investigated section and correlates with a low resistivity volume, interpreted as the fault zone conductor (Unsworth & Bedrosian, 2004).

Our retrieved *Vp* model has a very high similarity with seismic models computed with active seismic data, and computed absolute velocities are similar to sonic logs in the SAFOD well (Figures S6 and S9).

## 5 Velocity Gradients and Slip Behavior

The SAF near the SAFOD deep well is the perfect site for capturing mechanisms conditioning the fault slip behavior. To the south, the creep rate decreases and the fault ruptures with M6 earthquakes repeatedly during past centuries (Bakun & Lindh, 1985); the last two large ruptured the same segment in a complementary way (Custódio et al., 2005; Johanson et al., 2006). Several investigations were performed on the crustal structure of the SAFOD area, revealing the existence of large velocity contrasts across the SAF (Bleibinhaus et al., 2007; Hole et al., 2006; Li et al., 1997; Roecker et al., 2006; Ryberg et al., 2012; Thurber et al., 2004; Zhang et al., 2009). Although the existence of high velocity and lateral heterogeneities were related to difference in fault rheology (Eberhart‐Phillips & Michael, 1993), model resolution and a priori imposed velocity contrasts limited the recovering of along fault differences in material properties. Our solution, with an adaptive resolution‐controlled parameterization free of a priori contrasts, helps enhanced imaging of the entire seismogenic layer, with a resolution that previously was only prerogative of refraction profiles (Bleibinhaus et al., 2007; Ryberg et al., 2012). With respect to pioneering studies of the early 90s (see Eberhart‐Phillips & Michael, 1993), we retrieve and define more clearly the lateral heterogeneities across SAF.

**Figure 4:**
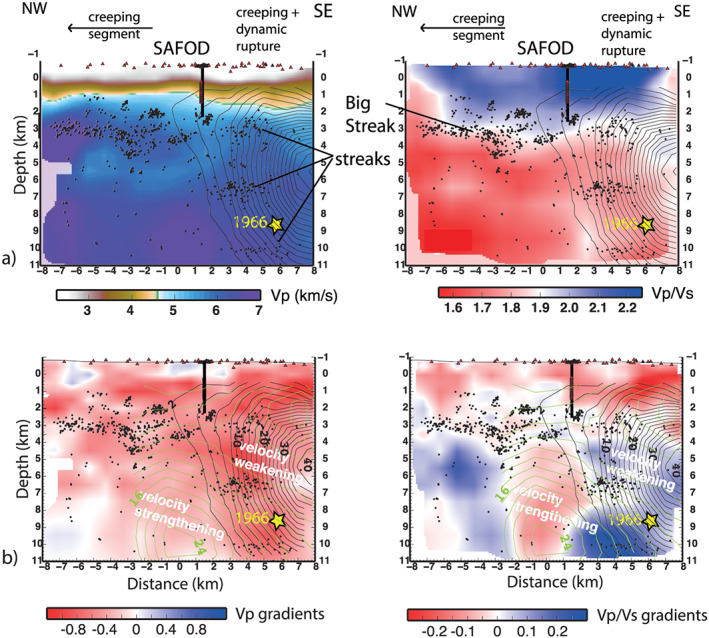
(a) *Vp* and *Vp*/*Vs* models and relocated seismicity along the SAF (profile “e” in Figure 1b). (b) *Vp* and *Vp*/*Vs* gradients along the SAF computed as the difference between profiles “f” and “d” in Figure 1. Earthquakes streaks identified by Waldhauser et al. (2004) and coseismic and postseismic slip in centimeter (black and green contours, respectively) of the 2004 earthquake from Johanson et al. (2006) are shown. Different velocity gradients are observed for the unstable slip section, identifying velocity‐weakening patch.

Generally, notable differences are visible for both *Vp* and *Vp*/*Vs* along the fault (Figure 4a) and between the NW (Figure 3a) and SE (Figure 3c) of the SAFOD area, with *Vp*/*Vs* ratio that changes from 1.75–1.9 to 1.5–1.75 to the NW. We compute velocity gradients across the SAF for the entire illuminated section from difference of velocities on the two sides of the fault (see traces in Figure 1b). The high‐resolution and small errors (less than 0.2 km/s and 0.08 for *Vp* and *Vp*/*Vs* gradients, see Figure S4) attest to the reliability of the observed gradients.

Along the fault, we observe a clear change in *Vp* and *Vp*/*Vs* gradients that coincides with the transition from the fault segments with different slip behavior (Figure 4b). *Vp* and *Vp*/*Vs* gradients are strong (up to 20% and 10% for *Vp* and *Vp*/*Vs*, respectively) in the section experiencing M6 shocks but negligible in the creeping segment (Figure 4b). The strongest *Vp* and *Vp*/*Vs* gradients are observed in coincidence with the hypocenter of the 1966 earthquake and the terminal part of the 2004 rupture. The coseismic slip of the 2004 mainshock (Johanson et al., 2006) correlates with high *Vp* contrasts and strong positive *Vp*/*Vs* contrasts (i.e., *Vp*/*Vs* is higher in the NA side; see Figures 3 and 4b). Conversely, postseismic slip dominates in portions with negative *Vp*/*Vs* contrasts and small *Vp* contrasts. Microseismicity clusters on the fault portion where the *Vp* and *Vp*/*Vs* gradients are strong (>0.6 km/s) and small, respectively (Figure 4b). Seismicity streaks observed along the SAF (Waldhauser et al., 2004 *)* are correlated with change in velocity gradients and with the main vertical *Vp*/*Vs* gradient, suggesting that they preferentially develop in zones of marked rheological transitions.

The different pattern of *Vp* and *Vp*/*Vs* anomalies (Figure 4a) and gradients (Figure 4b) between shallow and deep portions of the fault suggests different mechanisms of fault weakening. Drastic change of *Vp*/*Vs* coincides with the shallow seismic‐to‐aseismic transition. In the top of the SAF, high *Vp*/*Vs* anomalies and small *Vp*/*Vs* gradient that correlate with low resistivity (Unsworth & Bedrosian, 2004) are consistent with velocity‐strengthening behavior due to low frictional strength material in absence of significant across fault overpressure (i.e., absence of strong across‐fault velocity gradient), in agreement with laboratory strength measurements and observation relative to the shallow fault strands (Carpenter et al., 2015; Lockner et al., 2011; Moore & Rymer, 2007 *)*. In the deeper portion of the fault, velocity weakening and unstable slip occur in zone with strong velocity gradients (section 3 in Figure 4b). Low *Vp* and high *Vp*/*Vs* in the eastern side of the fault are consistent with the development of supra‐hydrostatic fluid pressure, potentially maintained by the SAF acting as a permeability barrier, as evidenced by regulations of deep fluid flux (Wiersberg & Erzinger, 2007 *)*. Conversely, fault weakening in the creeping section is not associated with enhanced fluid circulation around the fault (pervasive low *Vp*/*Vs*). Although the current resolution impedes discriminating thin layers of weak material in the fault core, the small velocity gradient is consistent with high elastic stiffness around the fault that inhibits the unstable slip behavior.

The correlation of across faults high velocity gradients with unstable slip behavior is an observation that at a large scale holds for other earthquakes (Ellis et al., 2017) but still needs further confirmation at a local high‐resolution scale.

## 6 Conclusions

We get high‐resolution images of the across‐faults seismic velocity gradients, along the key section of the SAF near SAFOD that exhibit changes in type of slip behavior. Such images are obtained, thanks to the application of a nonlinear tomographic method that resulted in data‐driven *Vp* and *Vp*/*Vs* models of the rock volumes around the SAF. Our result shows that slip rate variation and slip behavior correlate with these velocity gradients. At seismogenic depths, the across‐fault gradients are high in the fault section characterized by dynamic instability because of the greater attitude to develop transient supra‐hydrostatic fluid pressure. We highlight that weakening of shallow and deep portions of faults can be due to different mechanisms, where the effect of weak material (Carpenter et al., 2015) prevails at shallow depth. Although fault weakening can be related to the presence of weak material (Moore & Rymer, 2007) that could be thin enough to be not resolved by tomography, we hypothesize that across‐fault velocity gradients account for the different attitude to sustain fluid overpressure and change in elastic stiffness around the fault, conditions for the development of slip instability. Our results suggest that such gradients can be used to infer rock properties and fluid content and isolate fault portions able to support different slip mode.

## Supporting information

Supporting Information S1
