# Supplementary material for: Across‐Fault Velocity Gradients and Slip Behavior of the San Andreas Fault Near Parkfield
Source: Geophys Res Lett. 2020 Jan 17;47(1):e2019GL084480. doi: 10.1029/2019GL084480 (PMC7374945; doi:10.1029/2019GL084480)
Supplement: Supplementary file 1 — Supporting Information S1 [file GRL-47-e2019GL084480-s001.pdf]

Geophysical research Letter
Supporting Information for

Across-fault velocity gradients and slip behavior of the San Andreas fault near Parkfield

Piana Agostinetti N.[1], G. Giacomuzzi[2], and C. Chiarabba[2]

1 Department of Geodynamics and Sedimentology, University of Wien, Wien, 1010, Austria

2 Istituto Nazionale di Geofisica e Vulcanologia, Rome 00143, Italy

Contents of this file

Text S1 to S3
Figures S1 to S9

Introduction

In this file, we report the details of the analysis of the results of the tomographic inversion. In particular, we show the uncertainties on the investigated parameters. Moreover, we illustrate in details how our results compare with previous seismic models, sonic-logs and results from previous studies.

Text S1. Data selection, picking quality and double S-phases

In total we used 861 events, 72 blasts and 82 shots. The overall arrival time dataset consists of 43948 P-phase arrival times and 29158 Swave arrival times. For 18689 S-phases, two readings are observed as result of some anisotropy in the crust. When two readings are observed, only the arrival time with the lower picking error is included in the inversion. If the errors are identical, the earlier arrival time is chosen.

We derived data uncertainties from the SAC weighting scheme 0-4. We associated to these weights standard deviation of 0.025s (weight=0), 0.05s (weight=1), 0.1s (weight=2) and 0.3s (weight=3). No different weights are associated to earthquake and shot/blast data. With these data uncertainties, the differences between arrival times of the faster and slower S-phases are less than 1-sigma in the 95% of cases. Furthermore, we are confident that the effect of anisotropy on S-wave arrivals canb be considered as included in the picking errors.

Text S2. Model standard deviation and model reliability

We analyze and discuss the mean and standard deviation values of the 1D marginal PDF and compare them to the prior mean and standard deviation values. New information regarding the model parameter is added by the data to the prior information if the posterior standard deviation is less than the prior standard deviation. As described in Piana Agostinetti et al. (2015), the individual models are characterized by different Voronoi's tessellations and are all rasterized on the same regular grid (Figure S1). Mean and standard deviation values of the 1D marginal PDF over the velocity parameters (Vp and Vp/Vs ratio) are computed for each one of the grid nodes. Full maps showing the distribution of the standard deviations across the Vp and Vp/Vs ratio models are provided as supplementary Figures S2 and S3. As regard the event parameters, the prior standard deviations are as large as 1.0 km for Latitude and Longitude coordinates, and 2.0 km for depth, giving the possibility to the algorithm of moving the events across many nodes of the forward solver grid. The posterior standard deviations are less than the prior standard deviations for all events and all the hypocentral parameters. Posterior standard deviations are in the range 100-200 m for event latitude and longitude and a factor of two greater for event depth. About 80 earthquakes have posterior standard deviation greater than 500 m and are not shown in the Figures.
For any given velocity values, the posterior standard deviation can be quantitatively compared to the prior standard deviation. If the data constrained such parameter in some sense, the posterior standard deviation will be smaller than the prior one. In case of un-resolved parameters, the posterior standard deviation will be equal to the prior one. Since the prior standard deviation for Vp and Vp/Vs ratio are 0.5 km/s and 0.15, respectively, "resolved" portions of the investigated volume have posterior standard deviation for Vp (Vp/Vs ratio) lower than 0.5 km/s (0.15).

Text S2.1 Standard deviation of the Vp and Vp/Vs across-fault gradients

In Figure 4b, we reported the Vp (left) and Vp/Vs ratio (right) across-fault gradients, also called "velocity contrasts", computed as the difference in the elastic parameters along two vertical planes (spaced 1km from each other, traces "d" and "f" in Figure 1), that runs parallel to the local surface expression of the SAF. The geometry and position of the SAF discontinuity, needed to computed across-fault gradients, is defined here with a precision that accounts for the expected resolution of the velocity model (i.e. within 1 km). Additional tests have been done for increased or decreased spacing between the two vertical planes, without obtaining any significant difference. To better estimate the standard deviation of the velocity contrasts, for each point of the two vertical planes, we computed the difference in the velocity contrast in the original models sampled from the PPD and evaluate the standard deviation directly from such ensemble. The results are presented in Figure S4. Standard deviation is generally bounded between 0.1. and 0.2 km/s, for Vp contrast, and lower than 0.08, for Vp/Vs ratio contrast. These values are significantly smaller than the prior standard deviation which is expected to be twice the related standard deviation of the elastic parameters, namely 1.0 km/s and 0.3, for Vp and

Vp/Vs ratio contrasts, respectively. Thus, we assume that the data robustly constrain the across-fault velocity gradients along the portion of the SAF presented in Figure 4b.

Text S3. Comparison with borehole data

The Vp and Vp/Vs models around the SAFOD borehole are consistent with geological and borehole data (Figures S5 and S6), with absolute values that are slightly higher than borehole measurements. Standard deviations are in the ranges 0.15-0.25 km/s and 0.025-0.125 for the Vp and Vp/Vs, respectively. The excellent first-order correspondence between velocity models and sonic data confirm the capability to resolve fine details of the velocity model, without the need of strong a-priori constraints. The main Vp/Vs ratio pattern is well defined, with significant improvement respect to previous models. Near the borehole the resolution of the Vp and Vs models are different because both shot and blast data provide only P-wave arrival times.

Comparison with previous models

Local earthquakes tomography in the SAFOD and Parkfield areas has been applied several times with different inversion techniques, ray tracing algorithms and parameterization of the Earth structure (Lees and Malin, 1990; Michelini and McEvilly 1991; Eberhart-Phillips and Michael, 1993; Thurber et al., 2003).

Thurber et al. (2004) compared the results obtained with three different tomographic codes applied to the same dataset: Simul2000 (Thurber and Eberhart-Phillips, 1999), tomoDD (Zhang and Thurber, 2003) and tomoGRAD (Roecker et al., 2004; 2006). In all the tomographic codes the data inversion is carried out using a standard damped least squares technique. Some of these models include a velocity contrast of about 0.6 km/s across the SAF in the starting Vp model (higher than 10 %) and use borehole sonic-log measurements of Vp and Vs as strong or soft constraints on the inversion. A direct comparison with the more recent paper by Zhang et al. (2009) is more difficult because that study was done using more earthquakes and explosions than our model.

Despite the differences in ray-tracing, parameterization and treatment of earthquake locations, all models show first order similarity in the Vp structure. The main result is a sharp horizontal Vp contrast (up to 1km/s) across the SAF below 2-3 km depth. In most of the previous inversions this jump was partially related to the a priori constraint. A relative low Vp region down 4-8 km is found to the NE of the SAF and a relative high Vp body is found adjacent to the seismicity to the SW of the SAF, showing an offset about 4 km on the SW side of the SAF. Substantial differences appear only in the joint gravity-velocity model by Roeckers et al. (2004; 2006), which shows a velocity reversal to the SW of the SAF in between 3 and 5 km depth. In our model, both the velocity contrast across the SAF and the low Vp region to the NE of the SAF extend to a significantly greater depth.

Notable differences are present in the Vp/Vs models, maybe because of the different S-

wave data used and/or the different damping parameter used in the inversion. In Figure S7, we compare the Vp ad Vp/Vs models with those by Thurber et al. (2004). The models are derived by using the same starting earthquakes locations and ray-tracing parameters. In addition to the different inversion method, the main differences are the starting Vp model and the treatment of S-wave data. Thurber et al. (2004) use a 3D starting model with velocity fixed along the SAFOD pilot hole. This prevents the comparison with sonic-log data. Concerning the S-wave data, they use the whole S-wave dataset and invert P-S arrivals instead of S-wave arrivals. Instead, we use the S-wave arrivals with the lower picking errors, an approach similar to Roeckers et al. (2004; 2006). This can only partially explain the strong and notable differences between the Vp/Vs models.
The main differences between the two models (Figure S7) are: (i) the velocity reversal to the SW of the SAF, more evident in our model and consistent with gravity data, (ii) the increased resolution achieved with the adaptive parameterization, (iii) the Vp to the NE of the SAF, generally lower in our model and (iv) the deeper high and low Vp anomalies, more evident in our model, like. P-, S-wave and S-P residuals at the end of our inversion are smaller and symmetric (Figure S8) indicating the superior fit of data.

Comparison with active seismic models

Our retrieved Vp model has a very high similarity with the seismic model computed with active seismic data (Figure S9) and waveform inversion by Bleibinhaus et al. (2007). We compare the two models for the section across the SAF near SAFOD, and the agreement in velocity pattern and absolute velocity values is straightforward. In our case, we can reproduce the fine structure of the fault, with a resolution similar to that achieved with active seismic data, but with effectively much less costs.

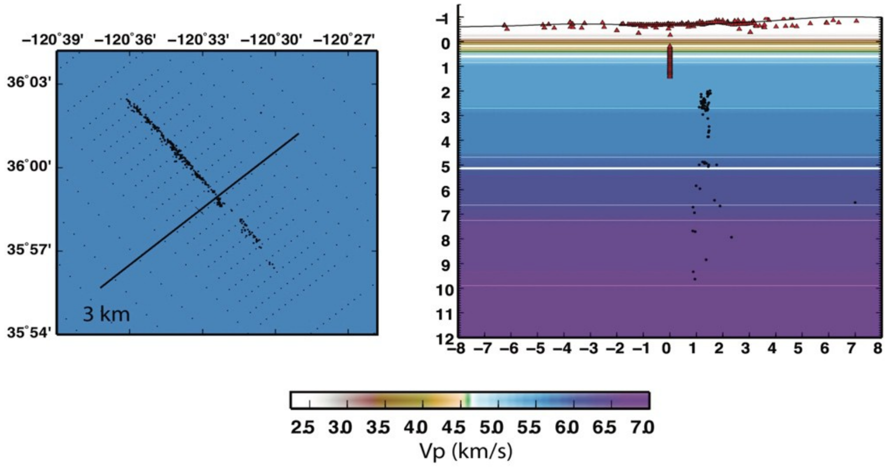

Figure S1. Prior mean Vp model plotted on a horizontal section at 3 km depth (left panel) and on a vertical cross-section (right panel). Red triangles: seismic stations, including the string of borehole receivers deployed in the SAFOD pilot hole; black circles: mean prior event locations.

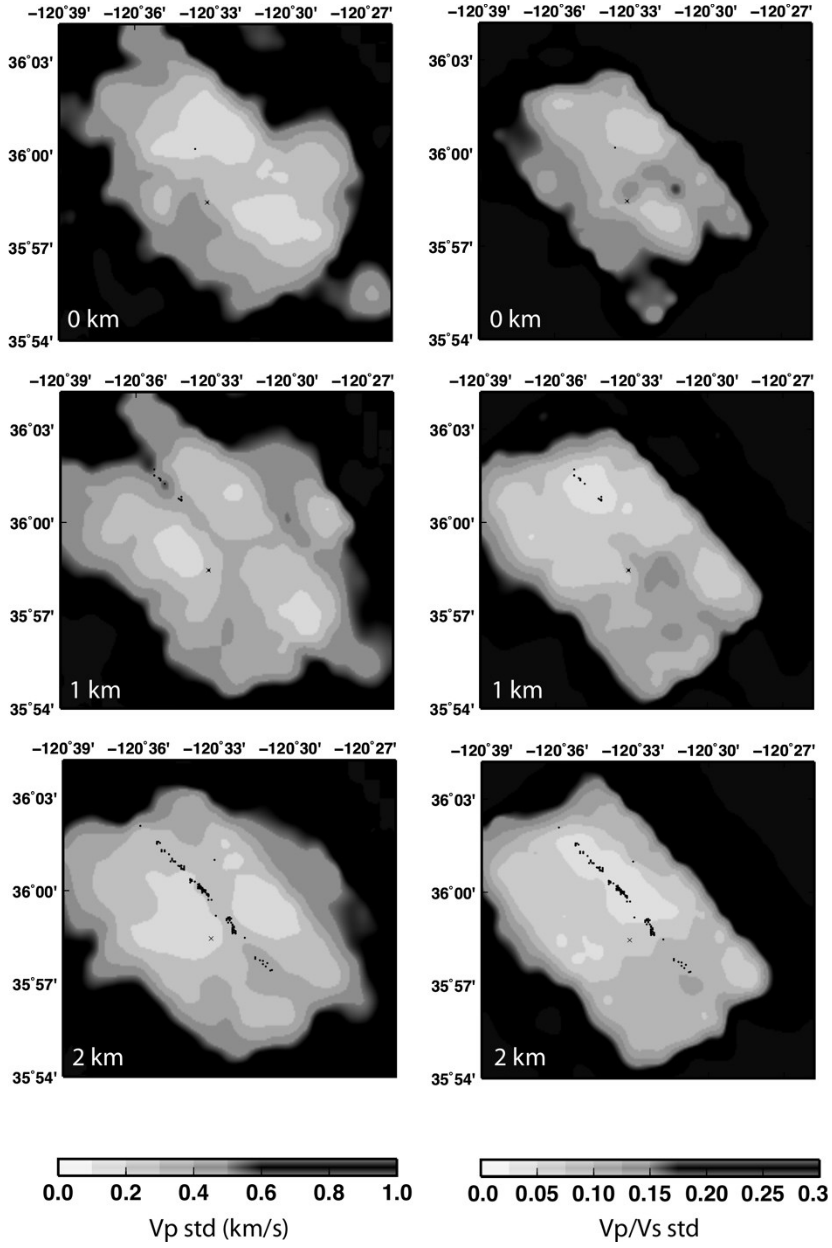

Figure S2. Standard deviations of the Vp (left panels) and Vp/Vs (right panels) at 0,1 and 2 km depth. Resolved portions of the investigated volume have posterior standard deviation for Vp (Vp/Vs ratio) lower than 0.5 km/s (0.15).

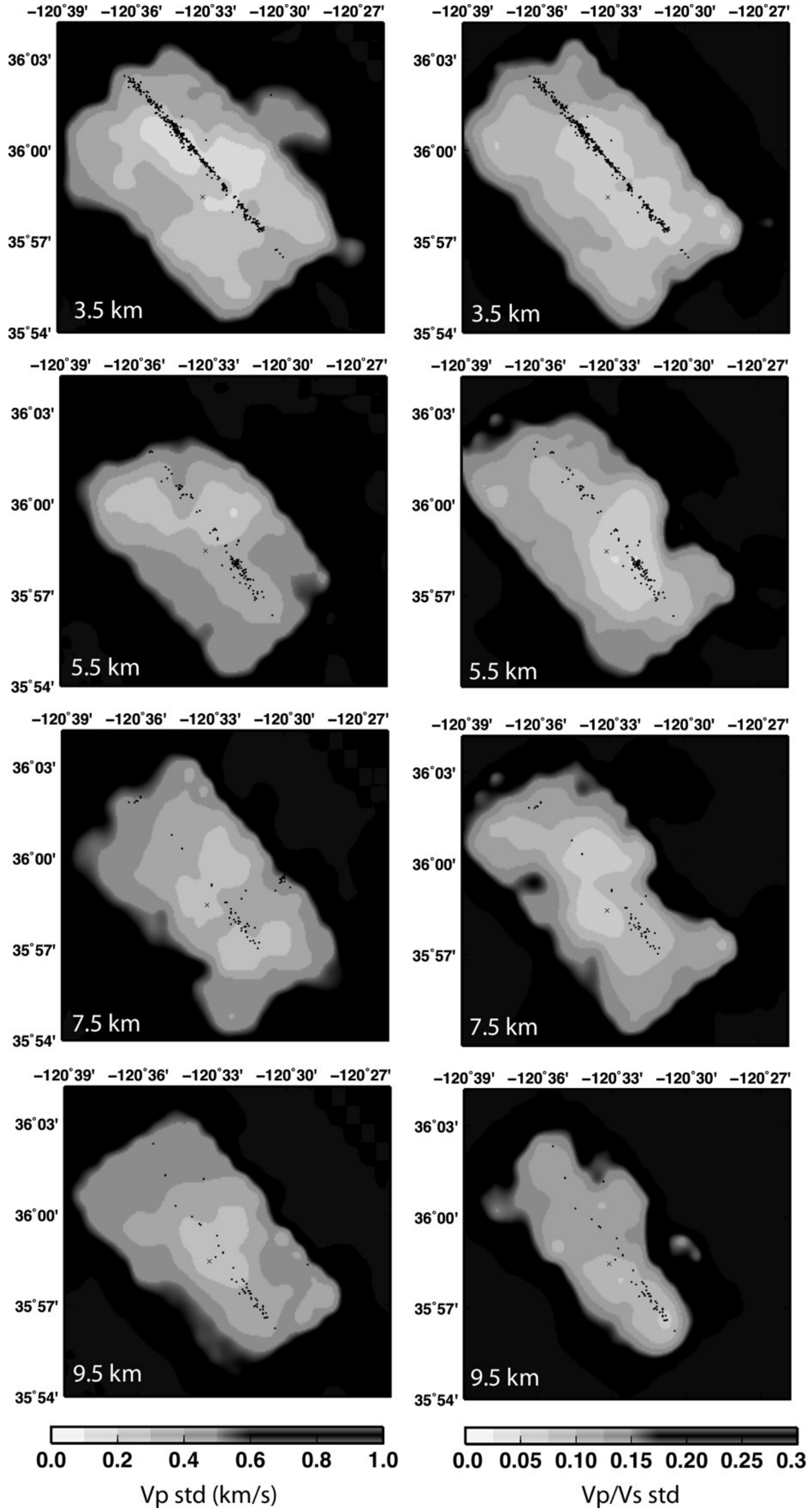

Figure S3. Standard deviations of the Vp (left panels) and Vp/Vs (right panels) between 3.5 and 9.5 km depth.

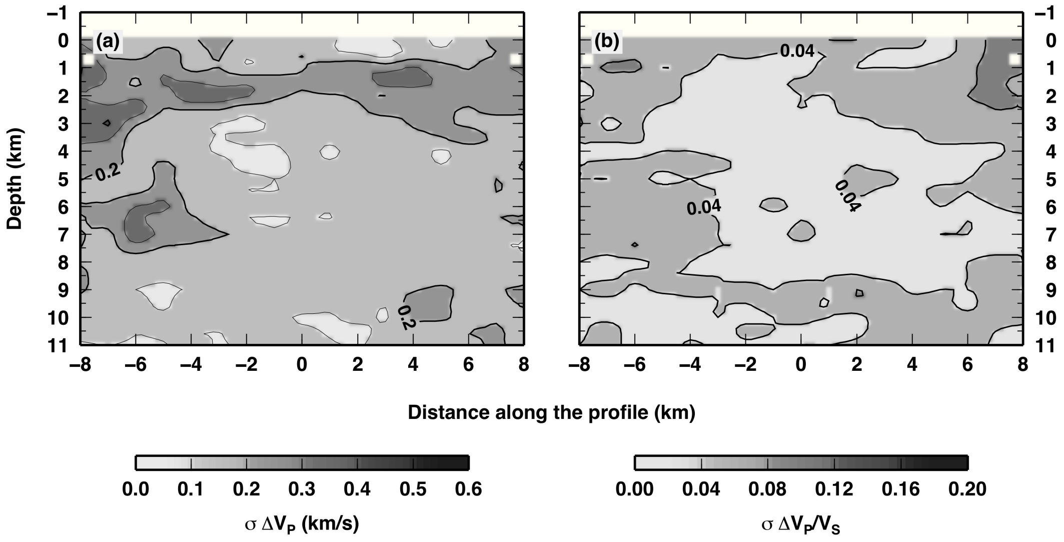

Figure S4. Posterior standard deviations of the Vp (a) and Vp/Vs (b) gradients across the faults, computed as the difference of the elastic parameters along two vertical planes on the two sides of the SAF (profile "d" and "f" in Figure 1).

a)
b)
c)

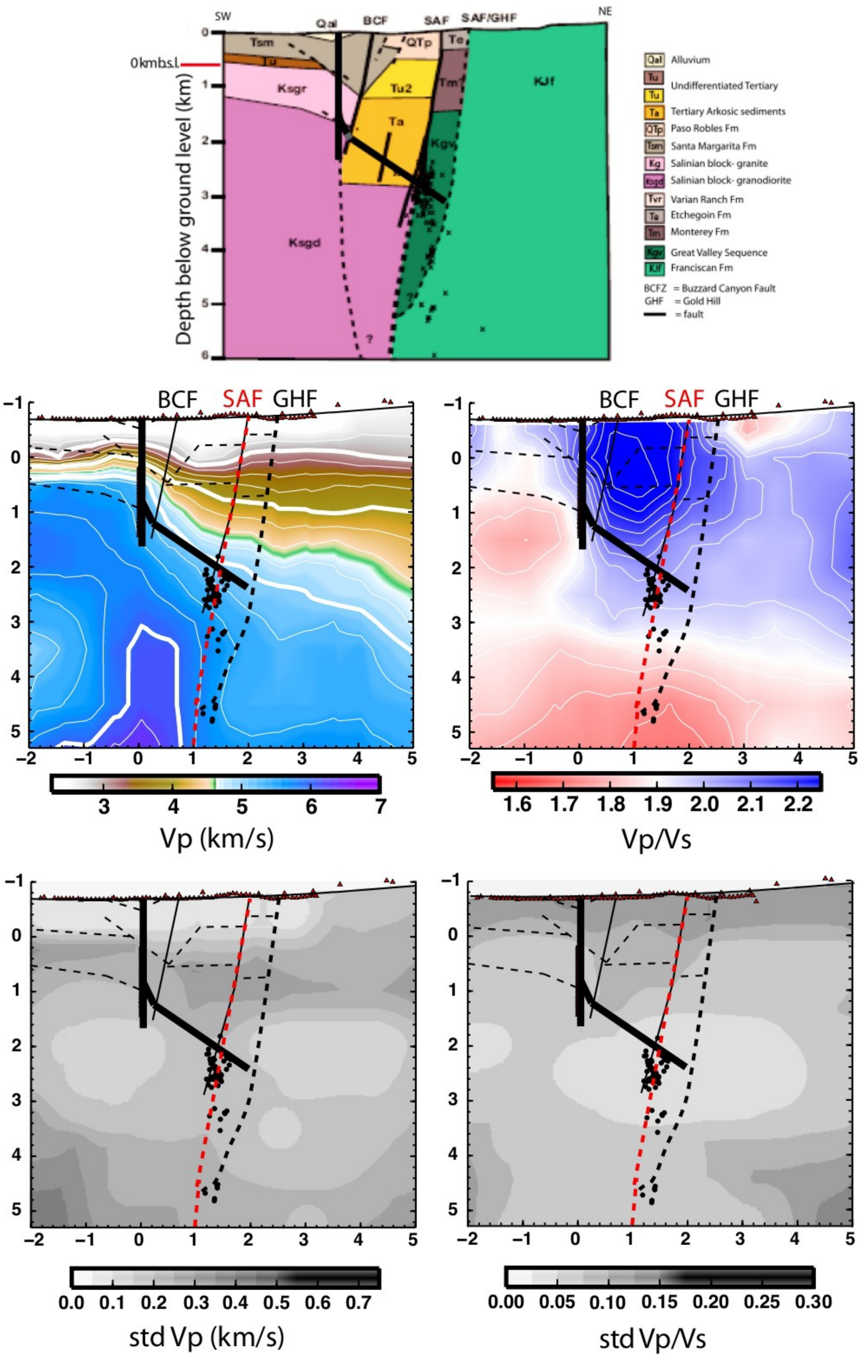

Figure S5. Comparison between Vp, Vp/Vs models and geology around SAFOD; a) geological section by Bradbury et al. (2007); b) velocity structure; c) error maps. Note the high resolution of velocities (std less than 0.3 km/s and 0.1 for Vp and Vp/Vs, respectively) across the SAFOD area.

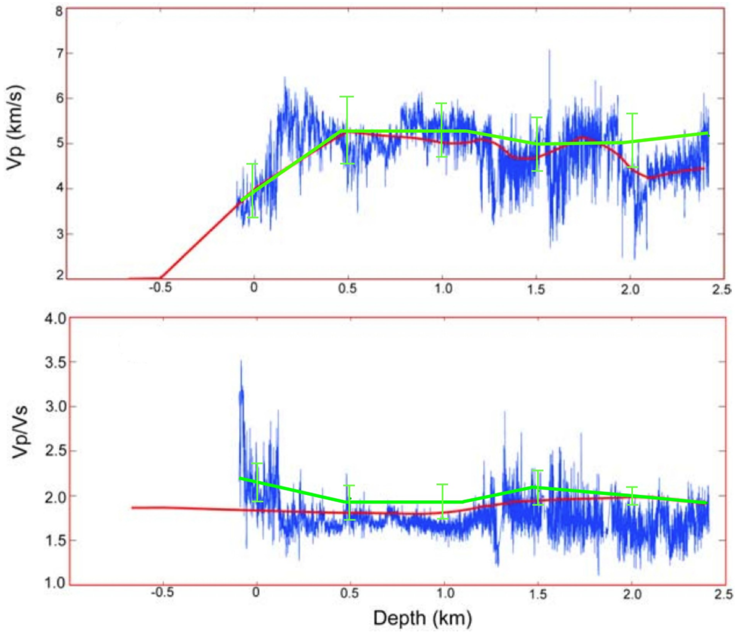

Figure S6. Borehole velocity logs in the main SAFOD hole (modified from Zhang et al., 2009); Vp and Vp/Vs values derived from our model (green curves) with vertical error bars equal to ±3sigma; red curves and from models by Zhang et al. (2009) (red curves). Note that the Vp values in Zhang et al. (2009) are a priori constrained with the values of velocity logs.

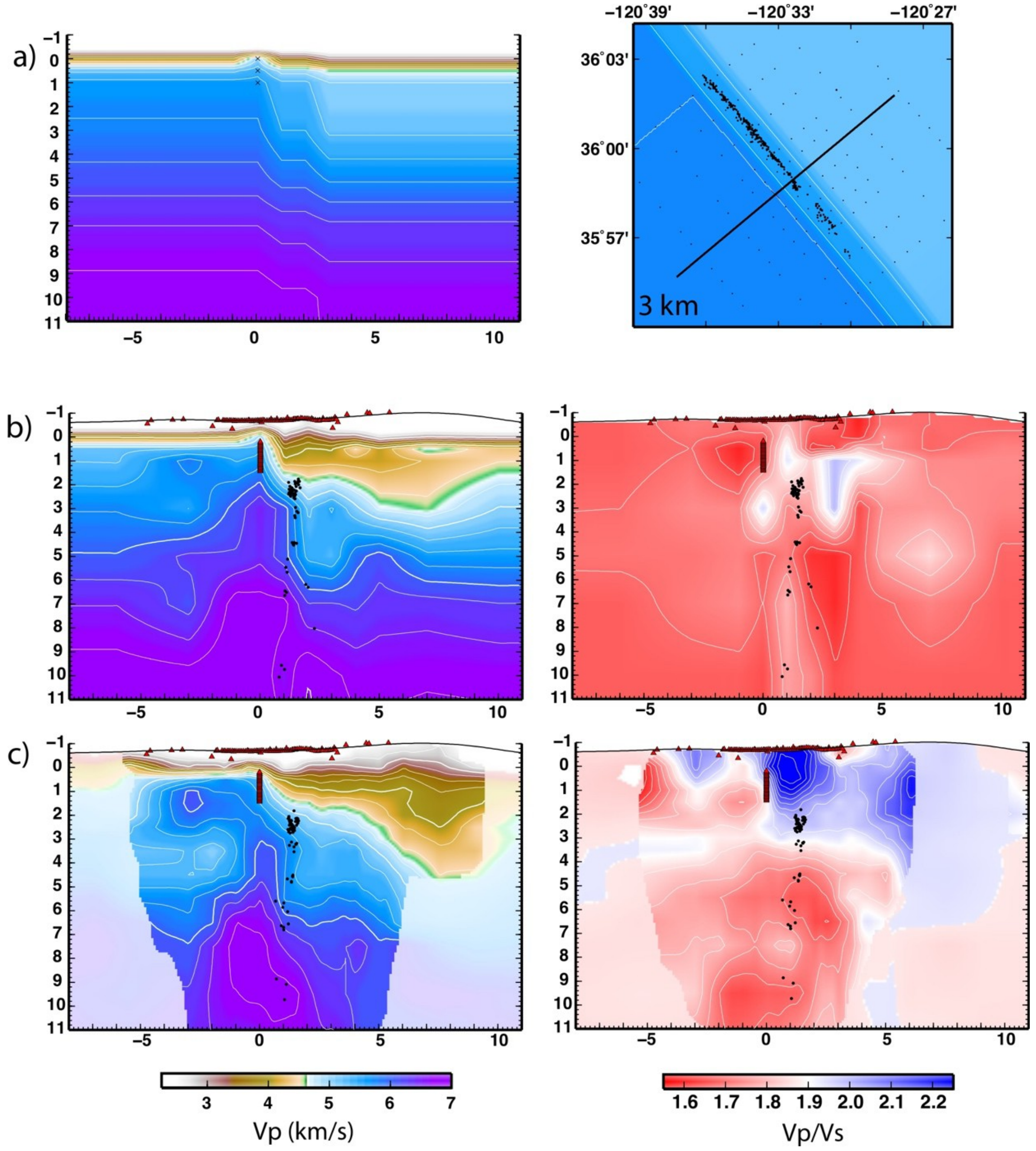

Figure S7. a) Starting Vp model by Thurber et al. (2004)); b) Vp (left panel) and Vp/Vs (right panel) final models by Thurber et al. (2004); c) non-linear Vp (left panel) and Vp/Vs (right panel).

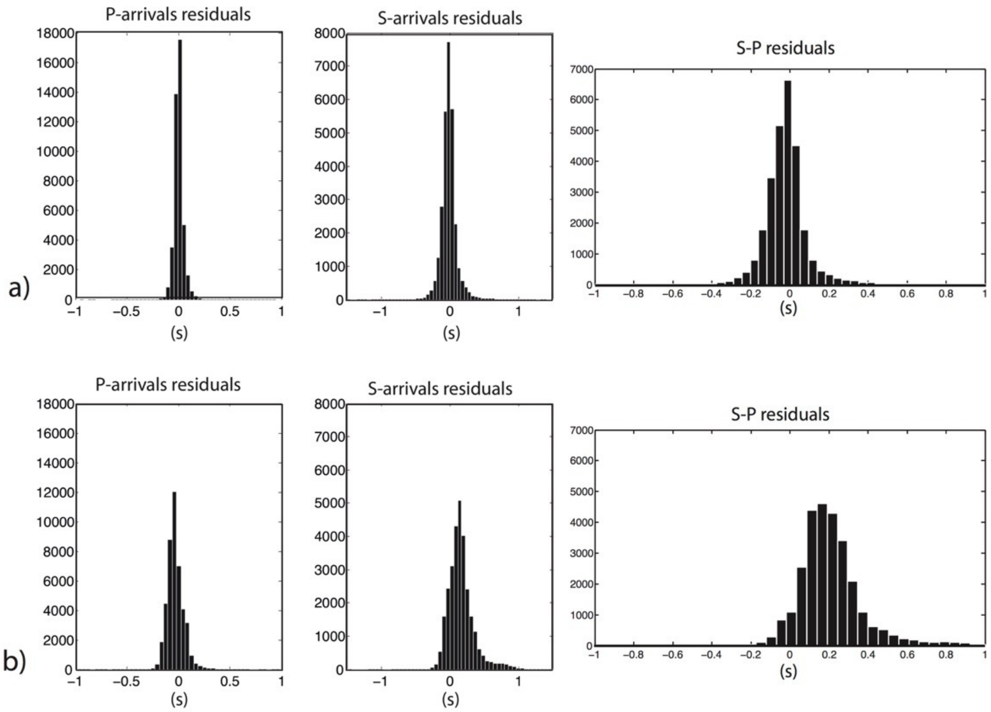

Figure S8. P- and S- residuals between observed and theoretical arrival times; computed in our model (a); computed in Thurber et al. (2004) model (b). S-P residuals between observed and theoretical arrival times; computed in our model (a); computed in Thurber et al. (2004) model (b).

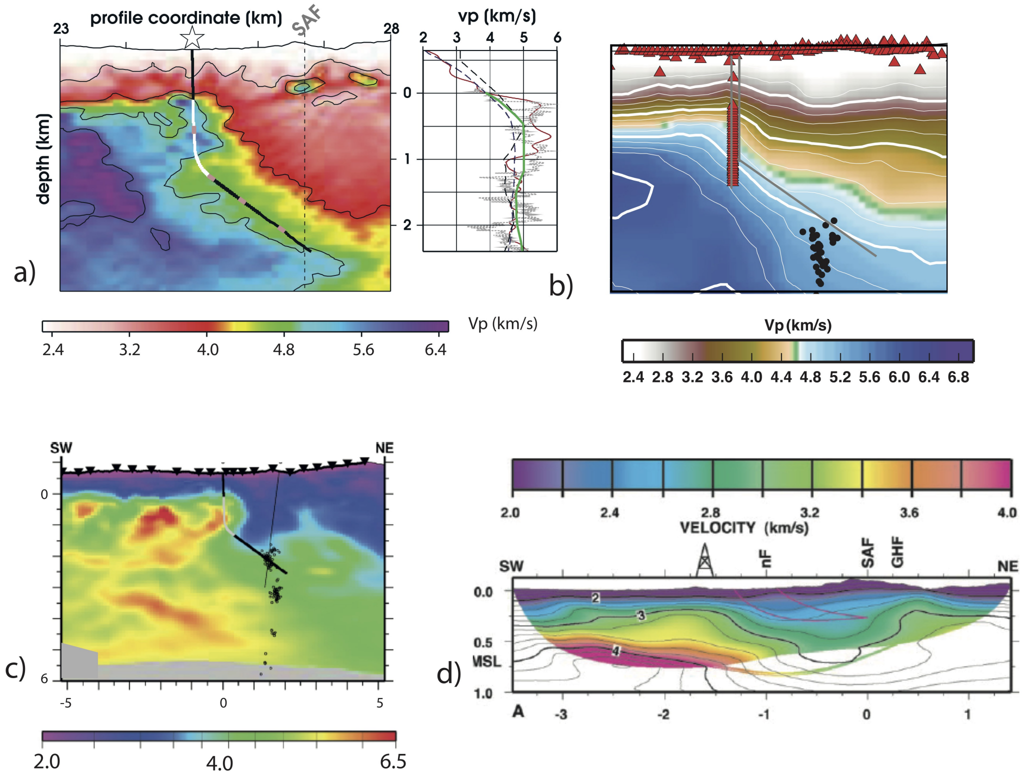

Figure S9. Comparison of the Vp model (panel b) with that derived from active seismic by Bleibinhaus et al. (2007) (a), Ryberg et al. (2012) (c) and Hole et al. (2001) (d). Note the excellent similarity in velocity pattern and absolute values. In the top central panel, the comparison of the velocity models along SAFOD: sonic log, grey dotted; Bleibinhaus et al (2007), red solid; Thurber et al. (2003, 2004), black dashed; our model, green solid. Panel "a" modified from Bleibinhaus et al (2007).
